# Cardiac Biomarkers for the Detection and Management of Cancer Therapy-Related Cardiovascular Toxicity

**DOI:** 10.3390/jcdd9110372

**Published:** 2022-10-29

**Authors:** Xinxin Zhang, Yuxi Sun, Yanli Zhang, Fengqi Fang, Jiwei Liu, Yunlong Xia, Ying Liu

**Affiliations:** 1 Department of Cardiology, The First Affiliated Hospital of Dalian Medical University, Dalian 116021, China; 2 Department of Cardiology, West China Hospital, Sichuan University, Chengdu 610041, China; 3 Department of Oncology, The First Affiliated Hospital of Dalian Medical University, Dalian 116021, China

**Keywords:** cardiac biomarker, cardiotoxicity, cardio-oncology, early detection

## Abstract

Cardiotoxicity is one of the major side effects of anti-cancer therapy affecting the overall prognosis of patients and possibly leading to the discontinuation of chemotherapy. Traditional cardiovascular tests such as electrocardiography and transthoracic echocardiography have limited sensitivity and specificity for the early detection of myocardial injury. Cardiovascular imaging generally detects cancer therapy-related cardiac dysfunction (CTRCD) at advanced stages, whereas biomarkers are inexpensive, easily detected, reproducible, and capable of detecting even minimal cardiomyocyte damage or mild hemodynamic fluctuations. The presence of circulating cardiac biomarkers has been investigated as early indicators of cardiotoxicity and predictors of subsequent CTRCD. Currently, the most frequently used cardiac biomarkers are cardiac troponin (cTn) and natriuretic peptides (NPs). This review presents the evidence gathered so far regarding the usefulness and limitations of cardiac biomarkers in the field of cardio-oncology.

## 1. Introduction

In recent years, modern medical technology has made huge progress in the treatment of malignant tumours. Consequently, the survival time of most cancer patients has been significantly prolonged, and many tumours exist in the form of chronic diseases. However, one of the most common factors to consider regarding anti-cancer therapy is the potential cardiovascular toxicity [1]. The 2022 ESC cardio-oncology guideline states that, unlike most classical cardiovascular diseases, cancer therapy-related cardiovascular toxicity varies with the characteristics of the oncology patient population, disease spectrum, antineoplastic therapy regimen, and many other factors, due to the uncertainty of the pathogenesis. The new guideline presents the concept of cancer therapy-related cardiovascular toxicity (CTRL-CVT) for the first time, providing clear and standardized definitions of cancer therapy-related cardiac dysfunction (CTRCD) /heart failure (HF) /cardiomyopathy, myocarditis, vascular toxicity, hypertension, arrhythmia, and QT interval prolongation [2]. The guideline also emphasizes the important role of cardiac markers in the early detection of cardiotoxicity. Cardiac biomarkers can be used at different stages of cancer treatment, for example, to assess baseline risk before starting cancer treatment, to monitor early toxicity during treatment, and to screen survivors for late side effects [3]. Over the past few decades, cardiac biomarkers have been investigated for identifying, assessing, and monitoring drug-induced cardiotoxicity at an early stage. Daniela et al. summarized the progression of cardiotoxicity resulting from anti-cancer therapy: as chemotherapy is initiated, troponin is released due to myocardial cell damage. Subsequent myocardial deformation causes a decrease in global longitudinal strain (GLS), and as asymptomatic cardiotoxicity progresses, left ventricular ejection fraction (LVEF) decreases; eventually, patients develop symptoms of HF [4]. Taken together, cardiac biomarkers should be integrated into the entire cancer treatment process to improve the early diagnosis of cardiovascular disease in cancer patients [5]. A guideline paper for serum biomarkers in cancer patients recommended that cardiac biomarkers be under surveillance before, during, and after cancer therapy with different cardiovascular risks [5]. However, the guideline did not provide any details regarding the appropriate management in the event that a biomarker is elevated. This review highlights the significance and management advice when biomarkers are abnormal during treatment with different anti-cancer therapies.

## 2. Baseline Assessment of Cardiovascular Risk Factors in Cancer Patients

Current oncology practice, including treatment planning and regimens for cancers with potential cardiovascular toxicity, provides a unique opportunity to fully assess cardiovascular health prior to the initiation of cancer therapies. In addition to classical cardiovascular risk factors, the characteristics of cancers, anti-cancer agents, and predictors of specific cardiovascular diseases (CVDs) should also be considered in the pre-treatment period. It is indispensable for all cancer patients planning to receive potentially cardiovascular toxic therapies to have a baseline assessment, including history collection, ECG, TTE, and cardiac biomarkers. NPs and cTn measurements at baseline are particularly important for evaluating the CVDs risk prior to anti-cancer therapy, as is the frequency of surveillance during and after cancer therapy [5]. The latest guideline recommends measuring baseline NP and/or cTn in all cancer patients at risk of CTRCD [2]. The HFA-ICOS Baseline Cardiovascular Risk Assessment Scale can be used to stratify risk based on specific anti-cancer drugs. Using this scale, cancer patients are classified to be at low, intermediate, high, or very high risks, which would facilitate personalized biomarker monitoring and treatment for them [6].

## 3. Classical Biomarkers of Myocardial Injury and Cardiac Dysfunction

### 3.1. cTn

Troponin is the most sensitive and cardiac-specific biochemical marker for detecting chemotherapy-induced cardiac injury. A troponin test is minimally invasive without direct damage to the patient, is less costly than echocardiography examination, and does not require special expertise [7]. The troponin complex, consisting of three subunits (troponin C, troponin T, and troponin I), is involved in the action of actomyosin leading to cardiac contraction and diastole. During cardiomyocyte necrosis, the cytoplasmic pool is rapidly depleted and the contractile apparatus ruptures, releasing large quantities of troponin T and I into the circulation. Thus, the presence of troponin T and I in peripheral blood indicates necrosis of cardiomyocytes (troponin C lacks specificity and is not appropriate for diagnosis) [8].

When the myocardium is injured, cTn is released into the blood and can be detected within 2–4 h (high-sensitivity cardiac troponin (hs-cTn) within 1 h), and reaches a peak concentration at 10–15 h. Depending on the degree of injury, it will return to the baseline level within 5–14 days. There will be a second peak in cTnT around 80 h after a large-area acute myocardial injury [9]. Persistent elevation in cTnI levels is generally associated with more severe CTRCD and higher rates of adverse cardiac events than transient elevation [10]. Notably, cTn is an organ-specific rather than a disease-specific biomarker [11].

To determine cardiotoxicity using hs-cTn, blood samples must be obtained before cardiotoxic drugs are administered. Moreover, during the entire treatment period, the changes should be monitored using the same hs-cTnI or hs-cTnT method [12] (Table 1). In general, an increase in hs-Tn concentration by 3–5 ng/L means 10–20 mg of myocardial tissue necrosis, which could not be detected by cardiac imaging techniques [13]. Troponin is, therefore, the most reliable biochemical marker for the early diagnosis of chemotherapy-induced cardiac injury [7]. Numerous studies have demonstrated that elevated levels of cTn can be detected prior to the irreversible CTRCD in cancer patients, highlighting the need for clinicians to optimize their treatment to prevent the onset and development of cardiotoxicity (Table 2).

### 3.2. NPs

There are three types of natriuretic peptides (NPs): atrial (ANP), cerebral (BNP), and type C (CNP), which are produced by the atria, ventricles, and endothelial cells, respectively. Among these markers, BNP is the most commonly used indicator to reflect intravascular and ventricular pressure and volume [30]. Volume or pressure overload induces the synthesis of pre-proBNP, which is processed to form proBNP. Under the action of endonuclease, proBNP is cleaved into BNP with biological activities such as natriuretic, diuretic, and vasodilator effects and inactive amino-terminal proBNP (NT-proBNP). BNP and NT-proBNP have similar clinical value for the evaluation of cardiac function, but with a few differences. NT-proBNP is more stable than BNP and does not appear to be affected by changes in anticoagulants, collection containers, body position, or circadian rhythm [31]. In patients with stable heart failure and other cardiac conditions (myocardial infarction, valvular heart disease, atrial fibrillation, or pulmonary embolism), NPs concentrations have a high prognostic accuracy for death and hospitalization for heart failure [32]. NPs are also an important screening tool for patients presenting with dyspnoea during anti-cancer treatment. NT-proBNP has been shown to have a high sensitivity (0.92) for the threshold value of 100 ng/L, but a low specificity for cardiotoxicity (0.5) due to other causes of increased NPs, such as atrial fibrillation and valvular heart disease [33]. A series of studies have demonstrated that NPs levels are important predictors of cardiovascular toxicity and are closely correlated with CTRCD (Table 3).

## 4. Role of Cardiac Biomarkers in Different Anti-Cancer Therapies

### 4.1. Anthracyclines

Anthracyclines exert cytotoxic effects by binding to the isoenzymes of topoisomerase 2 and increasing the release of reactive oxygen species (ROS). Both mechanisms are involved in the release of troponin from cell lysis [41]. Anthracycline-related cardiotoxicity can be detected earlier with troponins than with imaging methods [42]. In an early study, 703 cancer patients were measured for troponin I before, 3 days (early assessment), and 1 month (late assessment) after chemotherapy. In 70% of cases, troponin I levels were consistently within normal ranges, elevated only at early assessment in 21% of cases, and elevated at late assessment in 9% of cases. During the 3-year follow-up period, patients without TnI elevation showed no significant reduction in LVEF and had a low incidence of cardiac events (1%). In contrast, TnI-positive patients experienced a higher risk of major adverse cardiac events (MACE). Additionally, patients with persistent elevated TnI at late assessment were associated with more severe cardiac damage and a higher rate of adverse event compared to those with transient increase in TnI (84% versus 37%; *p* = 0.001) [10]. Thus, it is necessary to conduct more stringent surveillance for TnI-positive patients, particularly those with persistently high levels of TnI. Cardinale et al. conducted a study involving a total of 204 cancer patients receiving high-dose anthracycline chemotherapy. The enrolled patients were divided into TnI-positive and TnI-negative groups based on whether TnI exceeded the cut-off value at least once. There was a slight decrease in LVEF in both groups at the end of treatment, but this decrease was reversed only in patients who were TnI-negative [43]. In the adult population, patients who test positive for troponin are at a greater risk of CTRCD, and troponin has a high negative predictive value for future EF decline (93%) [44]. By analysing the predictive capacity of GLS combined with cTnT on cardiotoxicity, Kang et al. discovered a reliable and non-invasive method to predict cardiac insufficiency in patients receiving anthracycline chemotherapy (61% positive and 95% negative) [45]. Some other studies showed that mean peak serum troponin levels during chemotherapy were significantly lower in adult patients treated with β-blockers and angiotensin-converting enzyme inhibitors (ACEI) than in those who were not treated [44,46]. OVERCOME and PRADA studies have also demonstrated that the administration of cardioprotective drug therapies (ACEI and beta-blockers) is effective in preventing the development of cardiotoxicity-induced CTRCD in patients with increased troponin during chemotherapy [47,48].

In addition to troponin, NPs have been extensively studied as possible predictors or early detectors of anthracycline-induced cardiotoxicity, although their findings are less promising. NPs have not shown predictive value in the early-stage studies of cardiotoxicity caused by anthracyclines; however, promising studies in paediatric populations have emerged. It was found in a study of children treated with adriamycin for acute lymphoblastic leukaemia that NT-proBNP concentration was associated with an abnormal left ventricular thickness-to-dimension ratio, suggesting a detrimental effect on left ventricular remodelling during 4 years of follow-up (*p* = 0.01) [49].

Troponins and NPs are released into the circulation through different mechanisms, which makes their role as markers of cardiotoxicity different. Even in the absence of cellular death, troponin can be released into the circulation at an early stage of cellular damage. Nevertheless, the increase in blood NP levels is primarily dependent on atrial hemodynamic stress, which usually occurs when ventricular function is already significantly impaired [50]. In short, troponin provides greater insight into acute early cardiotoxicity in anthracycline treatment, while NPs may be more clinically relevant for late cardiotoxicity. The 2022 cardio-oncology guideline recommends the use of HF therapy, including ACEI/angiotonin receptor blocker (ARB) and beta-blockers in anthracycline-treated patients with elevated levels of troponin and NPs [2].

### 4.2. Trastuzumab

In recent studies, troponins have been analysed for their potential role in the early detection of myocardial damage in patients treated with novel targeted cancer therapies. Cardinale et al. examined TnI concentrations in 251 breast cancer patients who received trastuzumab. The study showed that troponin was elevated in 36 cases (14%), of which 62% developed CTRCD, compared to only 5% of patients with normal TnI (*p* < 0.001). Since none of the patients receiving trastuzumab without anthracyclines showed elevated TnI, the increase in serum markers may reflect anthracycline-induced cardiac injury promoted by trastuzumab, rather than de novo cardiac injury [15]. A study conducted by the HERA group demonstrated that baseline troponin elevation following anthracycline but before trastuzumab treatment was a powerful predictor of future trastuzumab-induced cardiotoxicity in women with reduced LVEF (hazard ratio 3.57–4.52) [22]. Morris et al. found that TnI increased prior to the decrease in LVEF in patients receiving trastuzumab and lapatinib after anthracycline chemotherapy [51]. This study provided novel evidence that biomarkers may be more sensitive than echocardiography examinations for the detection of myocardial injury in targeted therapies. Zardavas et al. also observed a correlation between high troponin levels at baseline and subsequent CTRCD in patients who opted for trastuzumab therapy. In addition, NT-proBNP appeared to have a higher sensitivity than troponins in detecting new-onset CTRCD during trastuzumab treatment [22]. In the latest guideline, HF therapy, which may include ACEI/ARBs and beta-blockers, is recommended for patients treated with HER2-targeted therapy who have elevated troponin and NP levels.

### 4.3. Immunotherapy

Immune checkpoint inhibitors (ICIs) activate the immune system by targeting immune checkpoints with antibodies, showing considerable clinical benefits in a wide range of cancers. However, the application of ICIs could cause immune-related adverse cardiac events, such as myocarditis, vasculitis, and pericarditis, as well as cardiac conduction disease and HF [6]. ICIs-related myocarditis may be mildly symptomatic at presentation, but it is highly lethal. Early diagnosis is therefore essential for timely intervention, even in the subclinical phase [52]. To date, the largest prospective study conducted by Waliany et al. in 2021 found that 11.2% of the 214 patients included had positive hs-TnI values (≥55 ng/L) during ICIs chemotherapy, whereas the incidence of myocarditis was only 1.4% (3 cases). Therefore, in most cases, positive hs-TnI was attributed to cardiovascular causes other than myocarditis [53]. A study by Mahmood et al. found that patients with a cardiac troponin level exceeding 1.5 ng/mL at discharge had a four-fold increased risk of MACE at follow-up. In order to monitor the response to ICIs therapy, continuous troponin measurements should be conducted during hospitalization and after discharge as well [25]. It has been reported that troponin measurements at baseline and weekly during weeks 1–3 can provide early detection of this toxicity associated with myocarditis [54]. Additionally, several clinical trials have shown that elevated NT-proBNP is very common (almost 100%) in ICIs-related acute myocarditis, but both NPs and troponins are of low specificity [55].

Chimeric antigen receptor (CAR-T) therapy, a new type of immunotherapy, involves genetically engineering T cells to target cancer cells. CAR-T therapy is used to treat a wide variety of haematological and solid cancers. One of the most significant side effects is cytokine release storm (CRS), which causes a severe systemic inflammatory response [56]. A study of 137 adult cancer patients treated with CAR-T showed that cardiac troponin is a sensitive biomarker for detecting cardiovascular toxicity [27].

### 4.4. Androgenic Deprivation

Campora et al. found that elevated NT-proBNP and TnT levels 3 months after the initiation of abiraterone were associated with a higher incidence of cardiac serious adverse events. Margel et al. observed that high levels of NT-proBNP and troponin at baseline predicted an increased risk of cardiovascular events within 12 months after GnRH agonist treatment, but not with GnRH antagonist therapy [57].

### 4.5. Radiotherapy

The effects of radiotherapy on the cardiac structure and function are significant, and radiation to the left breast or lung increases cardiovascular mortality and morbidity. Zaher et al. found that cardiac injury caused by radiotherapy for breast cancer was only observed in patients treated on the left side, with a small percentage of subjects experiencing a 20% or greater decrease in LVEF and a significant increase in biomarkers over the course of one year. Among patients receiving radiotherapy for left-sided breast cancer, TnI and creatine kinase isoenzyme levels were frequently elevated above the threshold [58]. A prospective study was conducted by Skyttä et al. on 58 patients with left breast cancer treated with radiotherapy and without chemotherapy. The study showed that the hs-cTnT elevated group received a markedly higher radiation dose than the non-elevated group. Moreover, there were no statistical differences between the two groups in terms of BNP levels [59]. However, in a follow-up study, the investigators discovered a remarkable increase in NT-proBNP levels 3 years after radiotherapy, suggesting that NT-proBNP may serve better as a marker for assessing long-term cardiac damage [60].

## 5. Management of Patients Receiving Potentially Cardiotoxic Treatments

**Figure 1:**
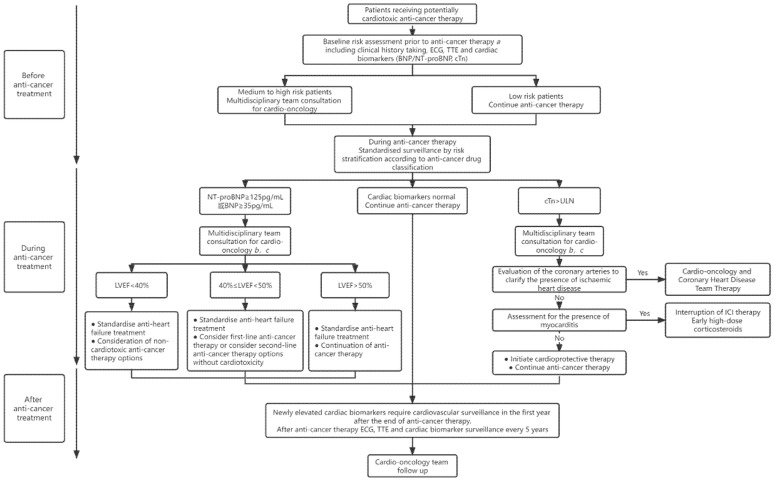
Management strategies for patients receiving potentially cardiotoxic anti-cancer therapy.

Based on several published guidelines, the Figure 1 illustrates a comprehensive approach to the surveillance and management of patients receiving cardiotoxic anti-cancer therapy [1,2,42,43,44]. For cases that develop cardiomyopathy or HF associated with anti-cancer therapy, a multidisciplinary discussion regarding the risk–benefit ratio of the interruption, continuation, or discontinuation of anti-cancer treatment is recommended [61]. Meanwhile, cancer survivors exposed to potentially cardiotoxic therapy should be monitored regularly over a long period of time, since HF may develop several years after cancer treatment [62].

In clinical practice, cardiac biomarkers can provide rapid risk stratification for patients with equivocal echocardiographic findings and help determine whether the symptoms are of cardiovascular origin [62]. In the presence of abnormal NPs levels, subsequent management strategies for cancer patients depend primarily on LVEF measurements. Unlike previous diagnostic criteria for cardiotoxicity, i.e., ≥10% reduction in LVEF and below 53% of the lower limit of normal LVEF in the absence of HF-related symptoms [45,46,47,48], the 2022 cardio-oncology guideline provides an updated definition of asymptomatic CTRCD and classifies it as mild (LVEF ≥ 50% and new relative decline in GLS by >15% from baseline and/or new rise in cardiac biomarkers); moderate (≥10% reduction in LVEF to 40–49% or LVEF <10% reduction to 40–49% and either relative decline in GLS by >15% or rise in cardiac biomarkers); or severe (LVEF reduction to <40%), which is consistent with the 2021 ESC HF guideline classification [2,62]. A prospective study involving a large cohort of patients showed that 82% of patients with CTRCD who were treated with ACEI and beta-blockers were able to recover and achieve normal EF at follow-up [63]. Data from the CECCY and PRADA trials revealed that receiving beta-blockers before and during anthracycline treatment reduced the levels of elevated troponin compared to placebo controls [23,64].

The new guideline states that in patients with elevated cTn, the onset of myocardial infarction and myocarditis needs to be considered, and imaging may be required if necessary [2]. Endomyocardial biopsy should be considered in those with suspected ICI-associated myocarditis if cardiac imaging or biomarkers cannot confirm the diagnosis [65]. Patients with confirmed ICI-associated myocarditis need to interrupt ICI therapy and receive high-dose corticosteroid therapy as soon as possible [2].

After anti-cancer treatment, new elevated cardiac serum biomarkers should be identified as risk factors in cardiovascular risk assessment, and cardiovascular surveillance is required for cancer survivors in the first year after the treatment ends [2]. In addition, cardiovascular surveillance is performed every 5 years for patients with established cardiotoxicity (high-dose anthracycline chemotherapy) [62].

## 6. Novel Biomarkers

Many studies have been exploring new biomarkers with the aim of identifying patients at risk of cardiotoxicity before, during, and after cancer treatment, providing early warnings, detecting subclinical cardiotoxicity, and developing novel cardiac risk assessments to guide clinicians in adjusting cardioprotective strategies in a timely manner. Some novel markers of myocardial ischaemia/necrosis, such as fatty acid binding protein (FABP) and glycogen phosphorylase isoenzyme BB (GPBB), have been reported. Myeloperoxidase (MPO) is a proatherogenic enzyme generated by neutrophils that promotes free radical production and lipid peroxidation [7]. MPO coupled with TnI assay was able to identify a subgroup of patients at an increased risk of cardiotoxicity, confirming the predictive value of this marker for the development of CTRCD [19]. Galectin-3, a biomarker relevant to cardiac remodelling and fibrosis, has been demonstrated to predict mortality in patients with acute or chronic decompensated heart failure [66].

## 7. Conclusions and Prospect

Future studies should look at patients with multiple baseline cardiovascular risk factors to identify those who might benefit from early prophylactic cardioprotective therapy. Novel and sensitive biomarkers will help predict cardiotoxicity and provide a basis for the further development of targeted preventive pharmacological strategies for CTRCD and cardiac complications.

## Figures and Tables

**Table 1:** Analytical characteristics of high-sensitivity cardiac troponin assays modified from Pudil R [5].

Company/Platform/ Assay	Cardiac Troponin Concentration		
	Cardiac Troponin Concentration	99th Percentile, ng/L	Kit Name
**hs-cTnI**			
Abbott Architect	1.1–1.6	26.2	Architect Stat High Sensitive Troponin-I
Beckman Access	1.0–2.3	17.5	Access hs-TnI
Siemens Centaur	1.6	47.3	High-Sensitivity TnI (TNIH)
Ortho Vitros	0.39–0.86	11.0	Hs-Troponin I
Quidel Triage True	2.33	27.9	Hs-cTnI/cTnI-II
**hs-cTnT**			
Roche Elecsys	5.0	14	hs-cTnT

**Table 2:** cTn is a predictor of CTRCD due to anti-cancer therapy.

Clinical Studies	Type of Treatment	Study Population	Age	Gender (M:F)	Pre-Treatment cTn Positivity Percentage (%)	Percentage of cTn Positivity After Treatment (%)	Mean cTn Level (Pre-Treatment)	Mean cTn Level (Post-Treatment)	cTn Threshold	Type of cTn Assay
Auner (2003) [14]	AC	Adults with haematological diseases	58	42:36	3/78 (3.8%)	12/78 (15.4%)	<0.01 ng/mL	40 (30–120) ng/L	30 ng/L	cTnT (Roche ElecIII TnT)
Cardinale (2004) [10]	AC	End-stage cancer, receiving high-dose chemotherapy	47 ± 12	216:487	0%	208/703 (30%)	NA	160 ± 240 ng/L	80 ng/L	cTnI (Stratus CS Tn I)
Cardinale (2010) [15]	Trastuzumab	Breast cancer patients receiving trastuzumab	50 ± 10	251 females	7/251 (2.8%)	36/251 (14.3%)	0.31 ± 0.45 ng/mL	0.31 ± 0.45 ng/mL	0.08 ng/mL	cTnI (Stratus CS, TnI)
Sawaya (2012) [16]	AC	Women with HER2-positive breast cancer receiving AC regimen chemotherapy, followed by paclitaxel and trastuzumab	50	81 females	NA	26/81 (32.1%)	1.3 pg/mL (0.7–6 pg/mL)	32 pg/mL (10–56 pg/mL)	30 pg/mL	cTnI (Siemens Healthcare Diagnostics, Tn I)
Draft [17] (2013)	AC	Patients with breast cancer, leukaemia, or lymphoma receiving low-to-moderate doses of anthracycline-based chemotherapy	50 ± 2	42:58	NA	26% of the participants met the criteria (0.06 ≤ x < 1.0 mg/mL)	0.02 ± 0.003 ng /mL	0.04 ± 0.01 ng/mL	0.06 ng/mL TnI	TnI (Beckman Coulter)
Ky (2014) [18]	AC	Breast cancer patients undergoing doxorubicin and trastuzumab therapy	50	78 females	NA		1.3 (0.7 to 4.0) μg/L	13.9 (2.6–31.6) μg/L	6.2 ± 13.7 μg/L	cTnI (Siemens Healthcare Diagnostics)
Putt (2015) [19]	AC	Breast cancer patients undergoing doxorubicin and trastuzumab therapy	49	78 females	NA	NA	1.30 (0.700–3.95) ng/L	3 months: 9.9 ng/L 6 months: 12.0 ng/L 9 months: 6.8 ng/L 12 months: 4.2 ng/L 15 months: 3.9 ng/L	3 ng/L	cTnI (Siemens Healthcare Diagnostics)
Olivieri (2017) [20]	AC	Lymphoma patients receiving AC or liposomal AC chemotherapy	60	56:43	4/99 (4%)	42/99 (42%)	NA	NA	30 ng/L	NA
Kitayama (2017) [21]	AC	Breast cancer patients receiving AC or trastuzumab	55–57	40 females	0%	4/40 (10%)	No cardiotoxicity: 0.007 ± 0.0017 ng/mL Cardiotoxicity: 0.005 ± 0.0019 ng/mL	0.044 ± 0.0109 ng/mL	0.04 ng/mL	Hs-cTnT (EcLusys high-sensitivity Troponin T, Roche Diagnostics)
Zardavas (2017) [22]	Trastuzumab	HER2-positive breast cancer patients receive neoadjuvant chemotherapy	50	452 females	cTnI: 56/412 (13.6%) cTnT: 101/407 (24.8%)	NA	cTnI: 28.4 ng/L cTnT: 13.3 ng/L	NA	cTnI: 40 ng/L cTnT: 14 ng/L	cTnI, hs-cTnT (Roche Diagnostics)
Gulati (2017) [23]	AC	Patients with early-stage breast cancer Stratification according to adriamycin dose ≥400 mg/m^2^ <400 mg/m^2^	≥400 mg/m^2^: 51 <400 mg/m^2^: 48.5	121 females	cTnI: 37/121 cTnT: 15/121	cTnI: 117/121 cTnT: 98/121	NA	NA	cTnI: 1.2 ng/L cTnT: 5 ng/L	cTnI:Architect i2000SR platform (Abbott Diagnostics, Abbott, IL, USA) cTnT:cobas 8000 e602 analyzer (Roche Diagnostics, Indianapolis, IN, USA)
Shafi (2017) [24]	AC	Patients with early breast cancer	47	82 females	0%	18/82 (22%)	NA	NA	NA	Stratus I
Mahmood (2018) [25]	ICIs	ICI-associated myocarditis cases	65	97:43	NA	94% of patients with myocarditis have elevated cTnT	1.18 (0.19–5.90) ng/mL	2.68 (0.24–7.63) ng/mL	NA	cTnT (4th generation Troponin T Assay)
Ponde (2018) [26]	Trastuzumab and/or lapatinib	HER2-positive early-stage breast cancer	50 (25–75)	280 females	cTnT: 0.6%	2 weeks: 0.6% Pre-surgery: 2.9%	NA	NA	cTnT > 0.015 μg/L	cTnT (Cobas Anti-Troponin T-Ak-Biotin/Anti-Troponin T-Ak-Ru)
Alvi (2019) [27]	CAR-T	137 patients with combined cancers receiving CAR-T treatment	62 (54–70)	93: 44	NA	29/137 (21.2%)	63 ng/L (34–110 ng/L)	63 ng/L (34–110 ng/L)	0.03 ng/mL	NA
Demissei (2020) [28]	Anthracyclines and trastuzumab chemotherapy	170 breast cancer patients receiving doxorubicin+ trastuzumab	43 (38–54)	170 females	NA	71/170 (41.8%)	3 ng/L	NA	14 ng/L	hs-cTnT: Cobas platform (Roche Diagnostics)
Finke (2021) [29]	Adjuvant or neoadjuvant	930 patients with combined cancers	61 (52, 70)	287:643	NA	11.4%	NA	NA	7 ng/L	Elecsys^®^ Troponin T high sensitive hs-cTnT assay (Roche Diagnostics)

**Table 3:** NPs are a predictor of CTRCD due to anti-cancer therapy.

Clinical Studies	Type of Treatment	Study Population	Age	Gender (M: F)	Pre-Treatment NPs Positivity Percentage (%)	Percentage of NPs Positivity After Treatment (%)	Mean NPs Level (Pre-Treatment)	Mean NPs Level (Post-Treatment)	NPs Threshold	Type of NPs Assay
Lee (2008) [34]	AC	86 patients with acute leukaemia, malignant lymphoma, or multiple myeloma	48.5 (20–65)	49: 37	NA	NA	25.0 pg/mL	305.8 pg/mL	100 pg/mL	Triage^®^ BNP kit
Lenihan (2016) [35]	AC	109 patients receiving anthracyclines	56	52: 57 (48%:52%)	1/109 (0.9%)	NA	NA	NA	100 pg/mL	NA
Palumbo (2016) [36]	AC	Female patient with left-sided breast cancer	63	78 females	NA	NA	14.66 ± 11.26 pg/mL	1 month: 23.96 pg/mL	6.7 ± 18.6 pg/mL	BNP (Shinoria-BNP)
Gulati 2017 [23]	AC	Patients with early-stage breast cancer Stratification according to adriamycin dose ≥400 mg/m^2^ <400 mg/m^2^	≥400 mg/m^2^: 51 <400 mg/m^2^: 48.5	121 females	NA	NA	BNP: 10.4 (5.0, 19.1) NT-proBNP: 48.3 (32.0, 76.5)	BNP: 12.0 (5.0, 23.0) NT-proBNP: 55.2 (29.5, 98.1)		BNP: Architect i2000SR platform (Abbott Diagnostics) NT-proBNP:cobas 8000 e602 analyzer (Roche Diagnostics)
Kitayama (2017) [21]	AC	Breast cancer patients receiving AC or trastuzumab	55–57	40 females	NA	no significant difference from baseline	19 ± 1.9 pg/mL	20 ± 6 pg/mL	18 ± 2.2 pg/mL	BNP (BNP-JP Architect)
Catino (2018) [37]	VEGF	Patients with metastatic renal cell carcinoma receiving sunitinib	62.5	56: 28	31.7 pg/mL	NA	31.7 pg/mL	NA	NA	BNP (Singulex single molecule counting assay)
Ponde 2018 [26]	Trastuzumab and/or lapatinib	HER2-positive early-stage breast cancer	50 (25–75)	280 females	NA	2 weeks: 5.8% pre-surgery: 12.1%		NA	NA	Anti-NT-proBNP-Ak~Ru(bpy)
Cornell (2019) [38]	Proteasome inhibitors	Carfilzomib or bortezomib for patients with multiple myeloma	66	63: 32	NA	NA	Carfilzomib: 24 pg/mL Bortezomib: 11 pg/mL	Mean rise in BNP of 165 pg/mL, mean rise in NT-proBNP of 5925 pg/mL	BNP: 100 pg/mL NT-proBNP: 125 pg/mL	NA
Bouwer (2019) [39]	Trastuzumab	Single-centre study, HER2-positive breast cancer patients	54	135 females	9%	NA	NA	NT-proBNP levels were slightly elevated from baseline levels (+2.9 pmol/L) in all individual patients	64 pmol/L	NT-proBNP level (Dimension Vista 500, Siemens Healthcare Diagnostics, Deerfield, IL, USA)
Blancas (2020) [40]	Trastuzumab	HER2-positive breast cancer patients	50.7 (26–76)	66 females	NA	NA	NA	320.6 pg/mL	<125 pg/mL (<50 years), <300 pg/mL (≥50 and 75 years) or <450 pg/mL (≥75 years)	NT-proBNP: ELISA (Roche Diagnostics^®^)
Finke (2021) [29]	Adjuvant or neoadjuvant	930 patients with combined cancers	61 (52, 70)	287:643	NA	17.2%	141 (70, 293.5)	NA	>300 ng/L	Stratus^®^ CS Acute Care™ NT-proBNP assay (Siemens AG, Berlin and Munich, Germany)

## Data Availability

Not applicable.
